# Mapping fMRI research in disorders of consciousness: a bibliometric study

**DOI:** 10.3389/fneur.2026.1807532

**Published:** 2026-05-04

**Authors:** Xi-Chen Wang, Di Zhu, Jun Lu, Guan-Lan Guo, Fan Fu, Lei Wang, Hong-Jian Lu, Bao-Guo Xu, Wei-Guan Chen

**Affiliations:** 1 Department of Rehabilitation Medicine, Nantong First People's Hospital, Nantong, Jiangsu, China; 2 Affiliated Teaching Hospital of Kangda College, Nanjing Medical University, Nanjing, China; 3 Affiliated Nantong Clinical College of Nantong University, Nantong, Jiangsu, China; 4 State Key Laboratory of Digital Medical Engineering, Jiangsu Key Laboratory of Robot Perception and Control, School of Instrument Science and Engineering, Southeast University, Nanjing, China

**Keywords:** application, bibliometrics, functional magnetic resonance imaging, prolonged disorders of consciousness, visual analysis

## Abstract

**Objective:**

To systematically map the evolution, academic network structure, and emergent research fronts of functional magnetic resonance imaging (fMRI) applications in prolonged disorders of consciousness (pDoC) through bibliometric analysis, with narrative contextualization of recent clinical trials.

**Methods:**

Bibliometric analysis was performed exclusively on the Web of Science Core Collection (WOSCC) database. A total of 418 articles and reviews (2009–September 2025) were analyzed using CiteSpace 6.3. R1 to visualize publication trends, country/institutional collaborations, journal co-citation networks, and keyword evolution. Separately, PubMed was searched (2009–September 2025) identifying recent clinical trials and therapeutic interventions; these 18 studies underwent narrative synthesis to supplement bibliometric findings with clinical context. No bibliometric analysis was performed on PubMed data.

**Results:**

A total of 418 publications from 39 countries or regions were identified in the field of fMRI for pDoC diagnosis. The United States contributed the highest number of publications (144), followed by China (99) and Belgium (1). Steven Laureys, Owen Adrian M, and Edlow Brian L were identified as the core scholars in this field. Co-citation analysis of journals revealed that NeuroImage was the most frequently cited journal for diagnosis-related research. Keyword clustering analysis identified core clusters directly related to diagnosis, including the Minimally Conscious State (MCS), resting-state functional magnetic resonance imaging (rs-fMRI), and scales.

**Conclusion:**

Research on fMRI for pDoC diagnosis from 2009 to 2025 has formed an academic network centered around the United States, China, and Belgium. Key research hotspots include the development of rs-fMRI diagnostic techniques, the identification of Cognitive-Motor Dissociation (CMD) and covert consciousness, and the screening of diagnostic biomarkers for pDoC subtyping. Future research should focus on multimodal approaches, such as parallel acquisition of fMRI-EEG–EMG, to establish closed-loop “consciousness-motor” biomarkers and conduct individualized intervention studies.

## Introduction

Prolonged disorders of consciousness (pDoC) are defined as a chronic condition of impaired consciousness persisting beyond 28 days after severe brain injury. Clinically, pDoC is classified into Vegetative State (VS) or Unresponsive Wakefulness Syndrome (UWS), Minimally Conscious State (MCS), and emergence from minimally conscious state (eMCS), reflecting a spectrum of residual cognitive and behavioral responsiveness (2, 3). Advances in critical and emergency care have markedly improved survival rates among high-risk patients, including those with cardiac arrest, traumatic brain injury, and stroke. Consequently, the prevalence of pDoC has risen steadily, with China alone accounting for an estimated 500,000 existing cases and 70,000–100,000 new cases annually (4). These patients typically remain bedridden and fully dependent on long-term care, predisposing them to severe comorbidities such as pneumonia, malnutrition, and pressure ulcers, thereby imposing a hefty socioeconomic burden-with annual direct medical costs in China estimated at 30–50 billion yuan (5).

Level of consciousness dictates clinical management, resource allocation, and even life-sustaining decisions, making accurate assessment critical. However, patients with pDoC often lack reliable motor or verbal output, leading to a misdiagnosis rate of up to 36–40% when relying solely on bedside behavioral scales such as the Coma Recovery Scale–Revised (CRS-R) (6, 7). Resting-state functional magnetic resonance imaging (rs-fMRI), a non-invasive technique requiring no active patient cooperation, offers high spatial resolution for mapping functional brain networks, which can detect covert consciousness when clinical assessment remain inconclusive, providing objective evidence to differentiate VS/UWS from MCS and identify Cognitive-Motor Dissociation (CMD) and has emerged as one of the most promising tools for precise assessment and individualized prognosis prediction in pDoC (8, 9).

fMRI, based on blood-oxygen-level-dependent (BOLD) signals, allows non-invasive mapping of neuronal population activity with millimeter-level spatial resolution (10). In the area of pDoC, fMRI is predominantly applied through three experimental paradigms: ① Active command-following paradigms employ motor imagery tasks-such as “imagining playing tennis” or “imagining swimming”-to detect volitionally driven brain activation; task-correlated responses in the premotor cortex or parahippocampal gyrus provide evidence of CMD, reducing diagnostic error rates from approximately 36–9% compared to behavioral assessment alone (11, 12). ② Passive sensory stimulation paradigms, including presentation of the patient’s own name or familiar auditory cues, probe the integrity of external information processing by examining activation gradients spanning primary auditory to higher-order semantic networks; the magnitude of such activation positively correlates with the likelihood of consciousness recovery at 12 months post-injury (13). ③ rs-fMRI quantifies static and dynamic connectivity within key neural circuits-such as the Default Mode Network (DMN), thalamocortical pathways, and cortico-striato-pallido-thalamo-cortical loops-enabling objective differentiation between VS/UWS and MCS. Beyond refining diagnostic precision, fMRI furnishes objective, reproducible neuroimaging biomarkers that not only help avert premature withdrawal of life-sustaining treatments but also inform personalized neurorehabilitation strategies and ethically grounded clinical decisions (14).

Despite growing research on the application of fMRI in the diagnosis and prognosis of pDoC, a systematic bibliometric analysis visualized using CiteSpace remains lacking for the recent three-year period (2022–2025). Existing reviews have predominantly focused on imaging paradigms or clinical validation, with limited quantitative examination of publication trends, emerging keywords, or co-citation networks within the “fMRI-pDoC” research domain (9, 15). CiteSpace, equipped with burst detection and co-citation clustering algorithms, enables precise identification of knowledge inflection points and emerging research hotspots. Although widely applied in studies on the evolution of neuroimaging methodologies, its use in the pDoC-fMRI research direction has yet to be explored. Therefore, this study employs CiteSpace for the first time to systematically visualize publication trends, country/institutional collaborations, journal co-citation networks, and emergent keywords in this field based on the WOSCC (2009-September 2025). To validate the clinical relevance of bibliometrically identified hotspots and capture emerging therapeutic interventions with insufficient citation history for burst detection, we conducted a targeted PubMed search (2022–2025) for high-grade clinical trial evidence. This sequential design-bibliometric pattern detection followed by clinical validation-follows convergent mixed-methods approaches in health sciences research.

## Method

### Study design

This is a bibliometric analysis mapping fMRI research in pDoC, with ancillary narrative review of recent clinical trials. Bibliometric analysis examines research structure and evolution through publication metadata, distinct from systematic review of clinical interventions.

### PICOS elements

Population: fMRI applications in pDoC (WOSCC *n* = 418; PubMed *n* = 18); Intervention: Diagnostic/prognostic fMRI; Comparator: Temporal evolution 2009–2025; Outcomes: Bibliometric metrics (citations, burst strength, betweenness centrality, cluster modularity) and trial quality grading; Study design: Articles/reviews (WOSCC); clinical trials (PubMed 2009–2025).

### Bibliometric analysis (WOSCC)

The literature for this study was systematically sourced from the WOSCC, a comprehensive and authoritative electronic database widely recognized for its high-quality scholarly resources. A search strategy combining controlled subject terms and free-text keywords was employed to ensure retrieval sensitivity and minimize omissions. To mitigate potential bias from periodic database updates, all searches were conducted and data were extracted on a single date: September 11, 2025.

Inclusion criteria were as follows: (1) publication types limited to articles or reviews; (2) publications written in English; (3) primary research involving human subjects.

Exclusion criteria comprised: (1) duplicate or retracted publications; (2) conference abstracts, book chapters, letters, editorials, and similar non-primary research materials; and (3) literature not relevant to fMRI in the diagnosis of pDoC.

Retrieved literature from WOSCC will be imported into EndNote, a reference management software. Two researchers will independently screen titles and abstracts to identify publications meeting the inclusion criteria. A third researcher will cross-check the screening results. Any disagreements will be resolved through discussion among all three researchers until consensus is achieved.

CiteSpace parameters: Time slicing January 2009–September 11, 2025 (1-year intervals); Top *N* = 50; g-index (*k* = 25); no pruning; Kleinberg burst detection (minimum 2 years); Freeman betweenness centrality (BC ≥ 0.1); LLR clustering. Cluster quality: Modularity *Q* = 0.623, Silhouette mean = 0.78.

### Narrative synthesis (PubMed)

Pubmed used as a complementary source to capture clinical trial publications and biomedical literature potentially missed by WOSCC, particularly for verification of emerging clinical trends. As this analysis involved only secondary use of previously published literature from these databases, no informed consent was required.

### Quality assessment

WOSCC: Citation-impact metrics (times cited, h-index, journal impact factor, network centrality). Risk-of-bias tools are not applicable to bibliometric analysis.

PubMed: GRADE-adapted grading for narrative synthesis. No formal risk-of-bias assessment as these trials were reviewed for trend contextualization, not systematic evidence evaluation.

## Results

### Annual number of publications from the WOSCC database

**Figure 1:**
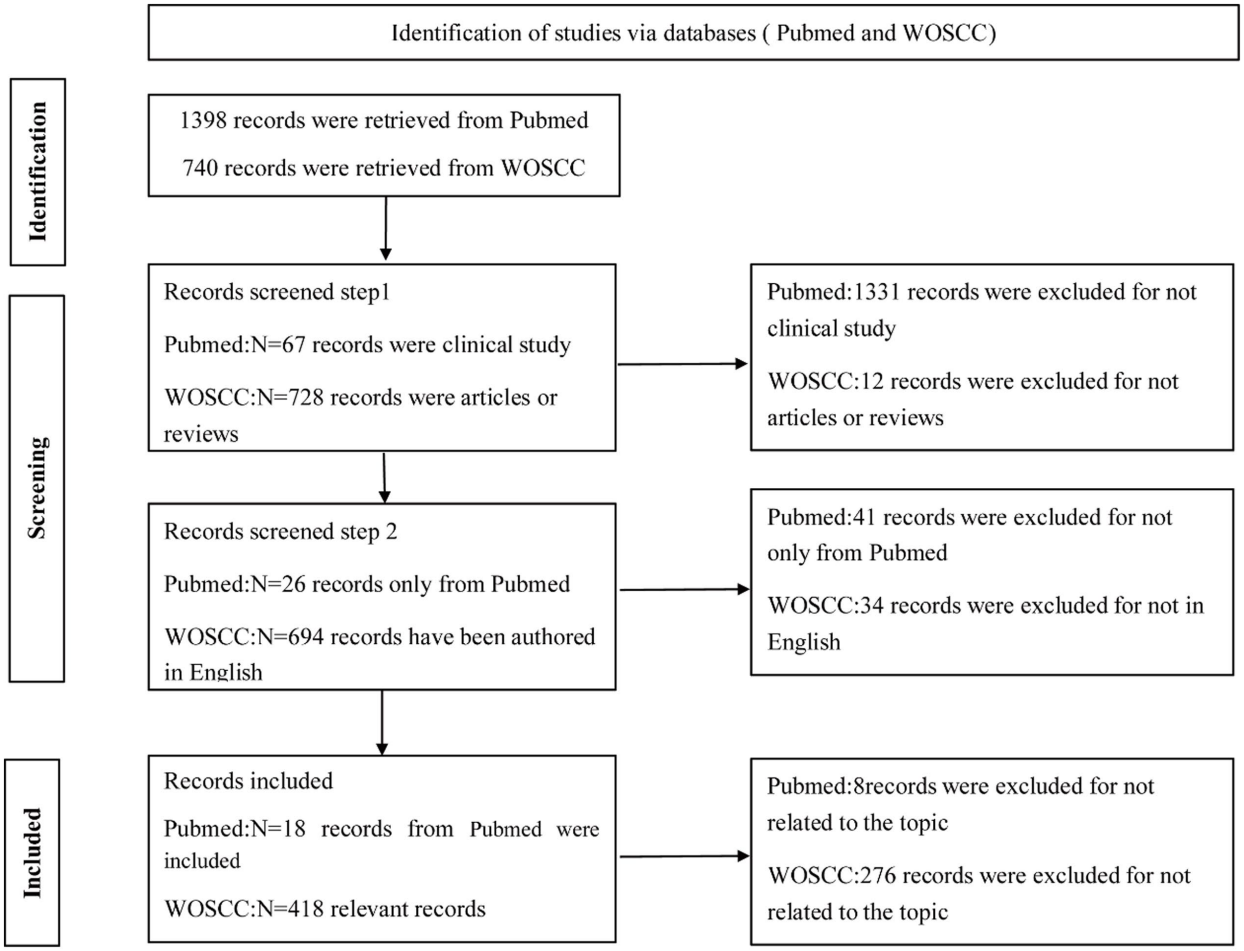
Bibliometric literature retrieval and screening protocol (Software: WPS Office 2023, Kingsoft, China).

**Figure 2:**
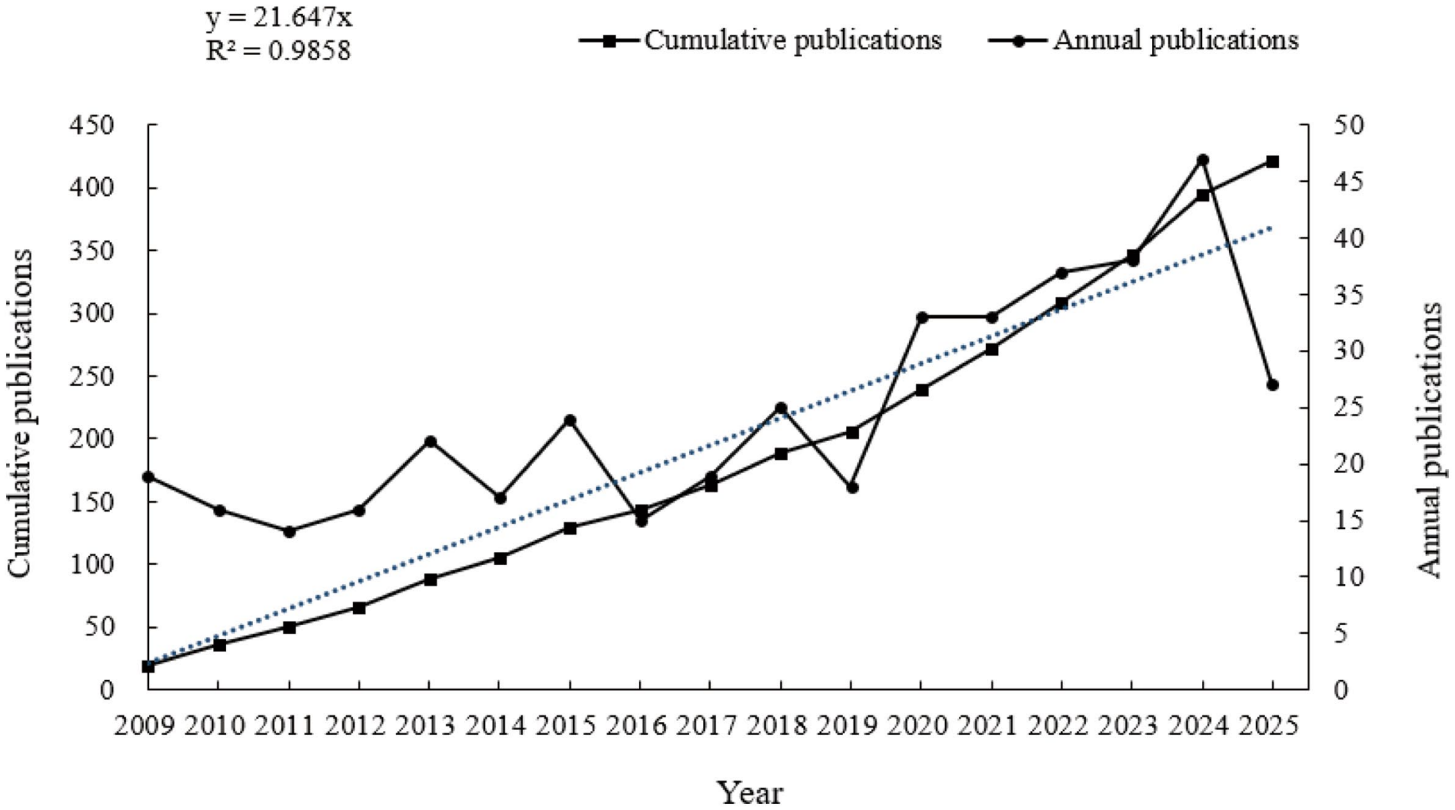
Annual and cumulative publications (Software: Excel 2023, Microsoft, USA).

From 2009 to September 2025, a total of 418 publications on the application of fMRI in the diagnosis of pDoC were identified in the WOSCC database (Figure 1). The annual number of publications showed an overall linear growth trend, reaching a peak of 47 in 2024 (Figure 2). As of September 11, 2025, 27 articles had already been published, with projections indicating that the total publication volume for 2025 is likely to reach a new peak by the end of the year.

### Analysis of country/region from the WOSCC database

**Figure 3:**
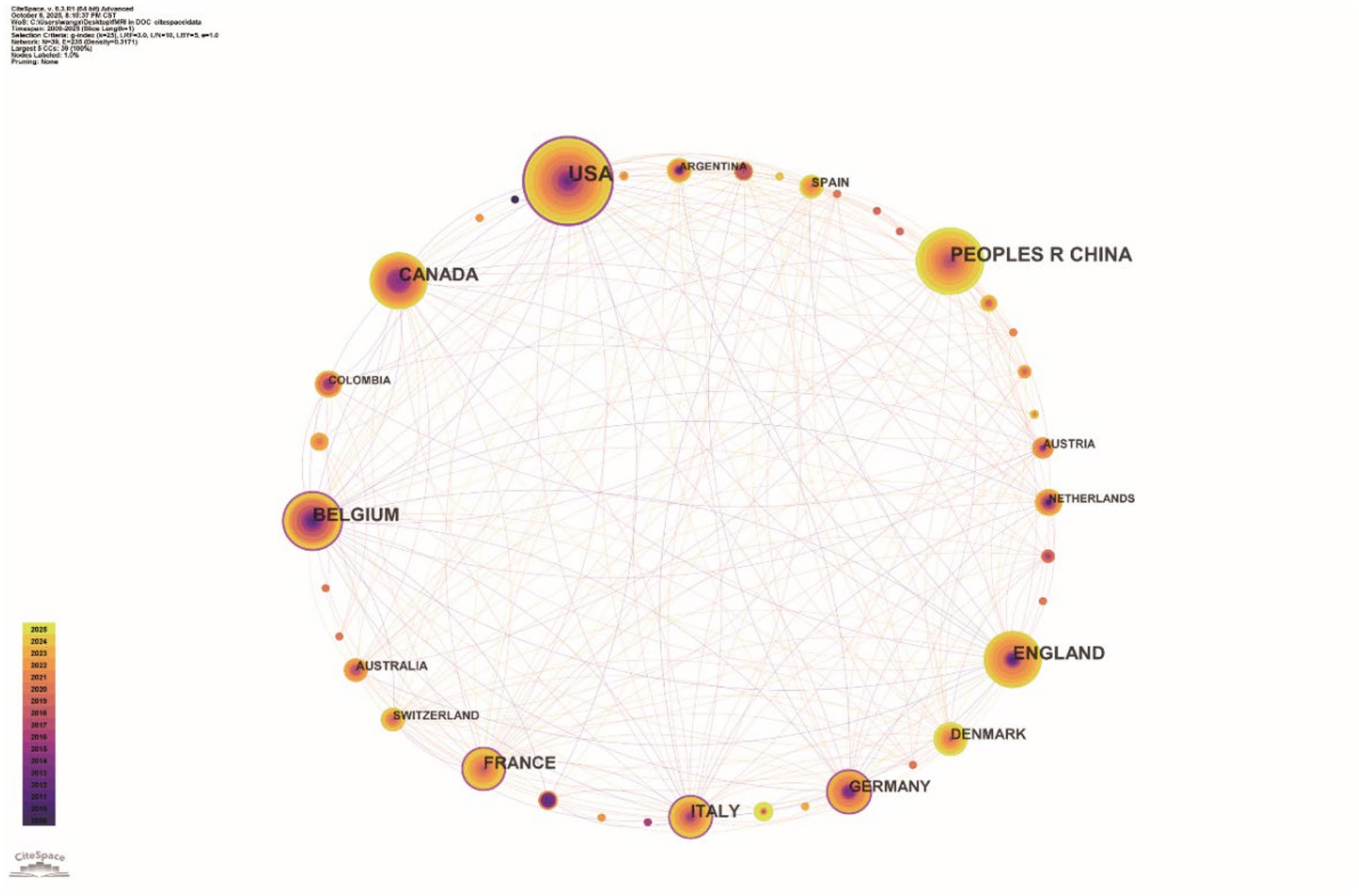
Country collaboration map time slice: January 2009 to September 2025, slice length: 1 year, node type: country, *N* = 39, *E* = 235. Above the image is the timeline (2009–2025) with colours. Node size is positively correlated with number of publications. Nodes with BC ≥ 0.1 show purple rings. Connecting lines indicate collaborations. (Software: CiteSpace 6.3. R1, Drexel University, Philadelphia, USA).

**Figure 4:**
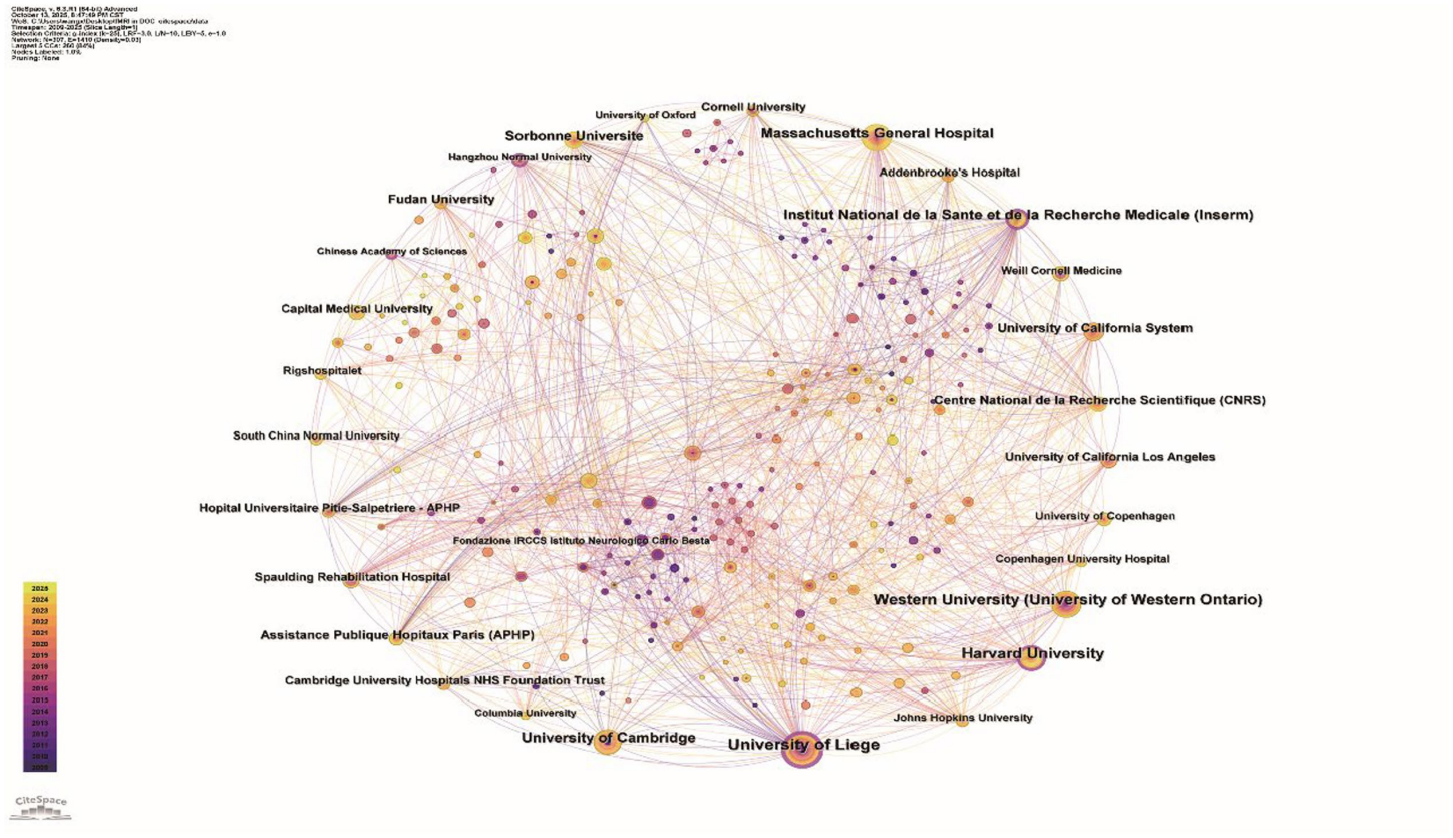
Institution collaboration map. Time slice: January 2009 to September 2025, Slice Length: 1 year, Node Type: Institution, Top *N* = 50, *N* = 307, *E* = 1,410. The colored time line (2009–2025) is shown at the top of the figure. Circles in the graph indicate the number of publications, larger circles indicate more publications by the institution, and connecting lines indicate the existence of collaborative or co-publishing relationships. Nodes with BC ≥ 0.1 are shown as purple rings (software: CiteSpace 6.3. R1, Drexel University, Philadelphia, USA).

A total of 39 countries or regions contributed to fMRI research on pDoC diagnosis. The United States led in publication output (144), followed by China (99), Belgium (1), the United Kingdom (1), and Canada (16). In the collaborative network analysis, node size corresponds to publication volume, and connecting lines represent direct international collaborations (Figure 3). Although Italy and France produced relatively fewer publications, they ranked highly in betweenness centrality (BC) (Table 1). Nodes encircled in purple indicate BC ≥ 0.1, signifying their role as bridges within the network—linking distinct research clusters and serving as pivotal hubs that help sustain the overall stability of the knowledge structure. The University of Liège ranked first globally with 69 publications and a BC of 0.35. Inserm (42, BC = 0.16) and Harvard University (44, BC = 0.13) also surpassed the BC threshold of 0.1. In Asia, Fudan University (21, BC = 0.10) was the first institution to enter the high-betweenness tier. All other institutions had BC values below 0.1 (Figure 4).

**Table 1:** Top 12 countries and institutions in fMRI in pDoC research.

Rank	Country	Count	BC	Rank	Institution	Count	BC
1	USA	144	0.26	1	University of Liège	69	0.35
2	China	99	0.02	2	Western University	49	0.07
3	Belgium	73	0.12	3	Harvard University	44	0.13
4	England	73	0.06	4	Institut National de la Sante et de la Recherche Medicale	42	0.16
5	Canada	70	0.10	5	Massachusetts General Hospital	36	0.06
6	Italy	53	0.18	6	University of Cambridge	34	0.06
7	France	52	0.11	7	Sorbonne Université	27	0.04
8	Germany	35	0.16	8	University of California System	25	0.03
9	Denmark	25	0.03	9	Centre National de la Recherche Scientifique	24	0.07
10	Australia	15	0.01	10	Assistance Publique Hopitaux Paris	22	0.03
11	Spain	15	0.02	11	Fudan University	21	0.10
12	Austria	14	0.02	12	Cornell University	20	0.06

BC: Betweenness centrality.

### Analysis of journals and references from the WOSCC database

Among the top 10 journals by co-citation frequency (Table 2), NeuroImage received the highest number of co-citations (359). Most leading journals originated from the United States and the United Kingdom, and all ranked within JCR Q1 or Q2 (impact factors and quartiles obtained from Clarivate’s Journal Citation Reports).

**Table 2:** Top 10 cited journals in fMRI in pDoC research.

Rank	Journals	Country	Count	BC	Year	IF (2024)	JCR
1	Neuroimage	USA	359	0.00	2009	4.7	Q1
2	Brain	England	352	0.01	2009	6.3	Q1
3	Neurology	USA	334	0.01	2009	9.9	Q2
4	Proceedings of the National Academy of Sciences of the United States of America	USA	276	0.01	2009	9.4	Q1
5	Human Brain Mapping	USA	256	0.01	2009	4	Q1
6	Science	USA	249	0.03	2010	56.9	Q1
7	The New England Journal of Medicine	England	246	0.02	2009	96.7	Q1
8	Archives of Physical Medicine and Rehabilitation	USA	246	0.03	2009	3.6	Q1
9	Lancet	England	244	0.04	2009	98.4	Q1
10	Annals of Neurology	USA	230	0.04	2009	8.3	Q1

BC: Betweenness centrality; IF: Impact Factor; JCR: Journal Citation Reports.

**Figure 5:**
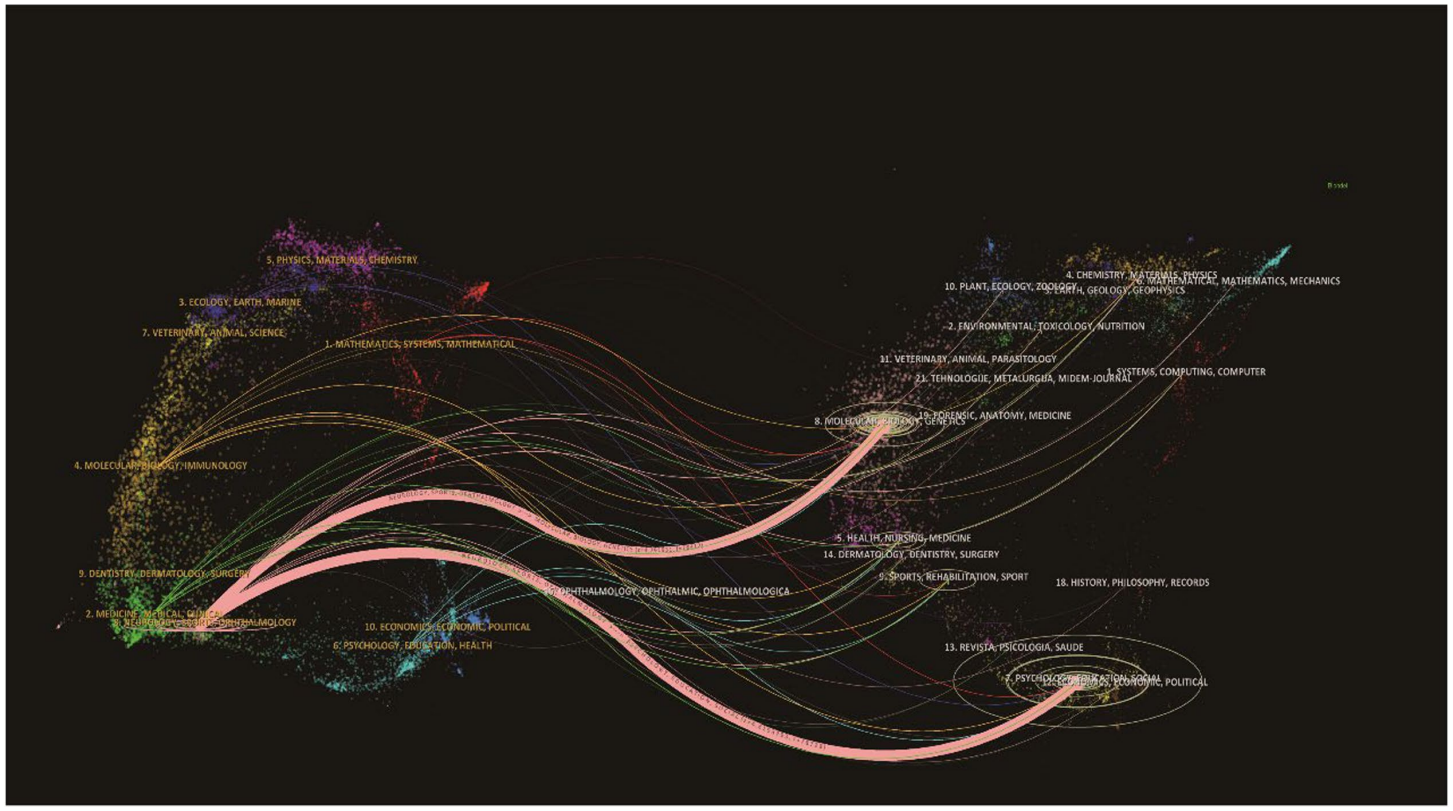
Dual-map overlaps of journals. The left side represents citing journals and the right side represents cited journals, with curve thickness indicating citation frequency. Four principal clusters constitute the core structure: “NEUROSCIENCES,” “SYSTEMS COMPUTING COMPUTER,” “MEDICINE GENERAL INTERNAL,” and supporting disciplines (e.g., “PSYCHOLOGY,” “BIOCHEMISTRY MOLECULAR BIOLOGY”). The pathway “NEUROSCIENCES → COMPUTER SCIENCE → CLINICAL MEDICINE” (*z* = 4.791931, *f* = 78,312) represents the dominant research trajectory, highlighting the bridging role of computational approaches between basic neuroscience and clinical medicine.

Dual-map overlay analysis of journals (Figure 5) reveals a multidisciplinary citation network, with citing journals on the left and cited journals on the right. The core structure comprises four principal clusters: “NEUROSCIENCES,” “SYSTEMS COMPUTING COMPUTER,” “MEDICINE GENERAL INTERNAL,” and supporting disciplines such as “PSYCHOLOGY” and “BIOCHEMISTRY MOLECULAR BIOLOGY.” The pathway “NEUROSCIENCES → COMPUTER SCIENCE → CLINICAL MEDICINE” (z = 4.791931, *f* = 78,312) emerged as the dominant research trajectory in the field.

The majority of the top 10 references are from JCR Q1 journals (Table 3). The most cited paper is “Willful modulation of brain activity in disorders of consciousness,” published in 2010 in The New England Journal of Medicine, with an impact factor of 96.7.

**Table 3:** Top 10 references ranked by co-cited frequency on fMRI in pDoC research.

Rank	Title	Count	BC	Year	First Author	Journal	IF (2024)	JCR
1	Willful modulation of brain activity in disorders of consciousness	66	0.02	2010	Monti M M	New England Journal of Medicine	96.7	Q1
2	Diagnosis and management of patients with disorders of consciousness	63	0.08	2021	Edlow B L	Nature Reviews Neurology	26.3	Q1
3	European Academy of Neurology guideline on the diagnosis of disorders of consciousness	58	0.02	2020	Kondziella D	European Journal of Neurology	5.1	Q1
4	An integrated multimodal approach to consciousness detection in the intensive care unit	50	0.08	2019	Demertzi A	Science Advances	11.7	Q1
5	Default network connectivity reflects the level of consciousness in vegetative state	50	0.08	2010	Vanhaudenhuyse A	Brain	11.3	Q1
6	Diagnostic accuracy of the vegetative and minimally conscious state: validation of the Coma Recovery Scale-Revised	45	0.02	2009	Schnakers C	BMC Neurology	2.8	Q2
7	Diagnostic precision of PET and fMRI in disorders of consciousness	40	0.06	2014	Stender J	Lancet	98.4	Q1
8	Detection of brain activation in unresponsive patients with acute brain injury	39	0.06	2019	Claassen J	New England Journal of Medicine	96.7	Q1
9	Detecting awareness in the vegetative state	35	0.01	2006	Owen A M	Science	56.9	Q1
10	Practice guideline update: disorders of consciousness	34	0.04	2018	Giacino J T	Neurology	9.9	Q1

BC: Betweenness centrality; IF: Impact Factor; JCR: Journal Citation Reports.

### Analysis of authors from the WOSCC database

A total of 474 scholars participated in fMRI studies related to pDoC diagnosis. As shown in Table 4, the top three authors ranked by number of published articles were Steven Laureys (17), Owen Adrian M (18), and Edlow Brian L (19). The top three most cited authors were Giacino Joseph T (306), Steven Laureys (241), and Monti Martin M (201).

**Table 4:** Top 10 authors and cited author in fMRI in pDoC research.

Rank	Author	BC	Count	Cited Author	Count	BC
1	Laureys S	0.09	50	Giacino J T	306	0.01
2	Owen A M	0.07	32	Laureys S	241	0.00
3	Edlow B L	0.01	23	Monti M M	201	0.01
4	Soddu A	0.01	18	Owen A M	195	0.01
5	Gosseries O	0.05	15	Boly M	189	0.02
6	Demertzi A	0.01	13	Schnakers C	182	0.03
7	Boly M	0.03	13	Schiff N D	180	0.01
8	Vanhaudenhuyse A	0.01	12	Vanhaudenhuyse A	161	0.01
9	Fernandez-espejo D	0.02	12	Demertzi A	159	0.07
10	Thibaut A	0.02	12	Fernandez-espejo D	137	0.03

### Analysis of keyword from the WOSCC database

**Figure 6:**
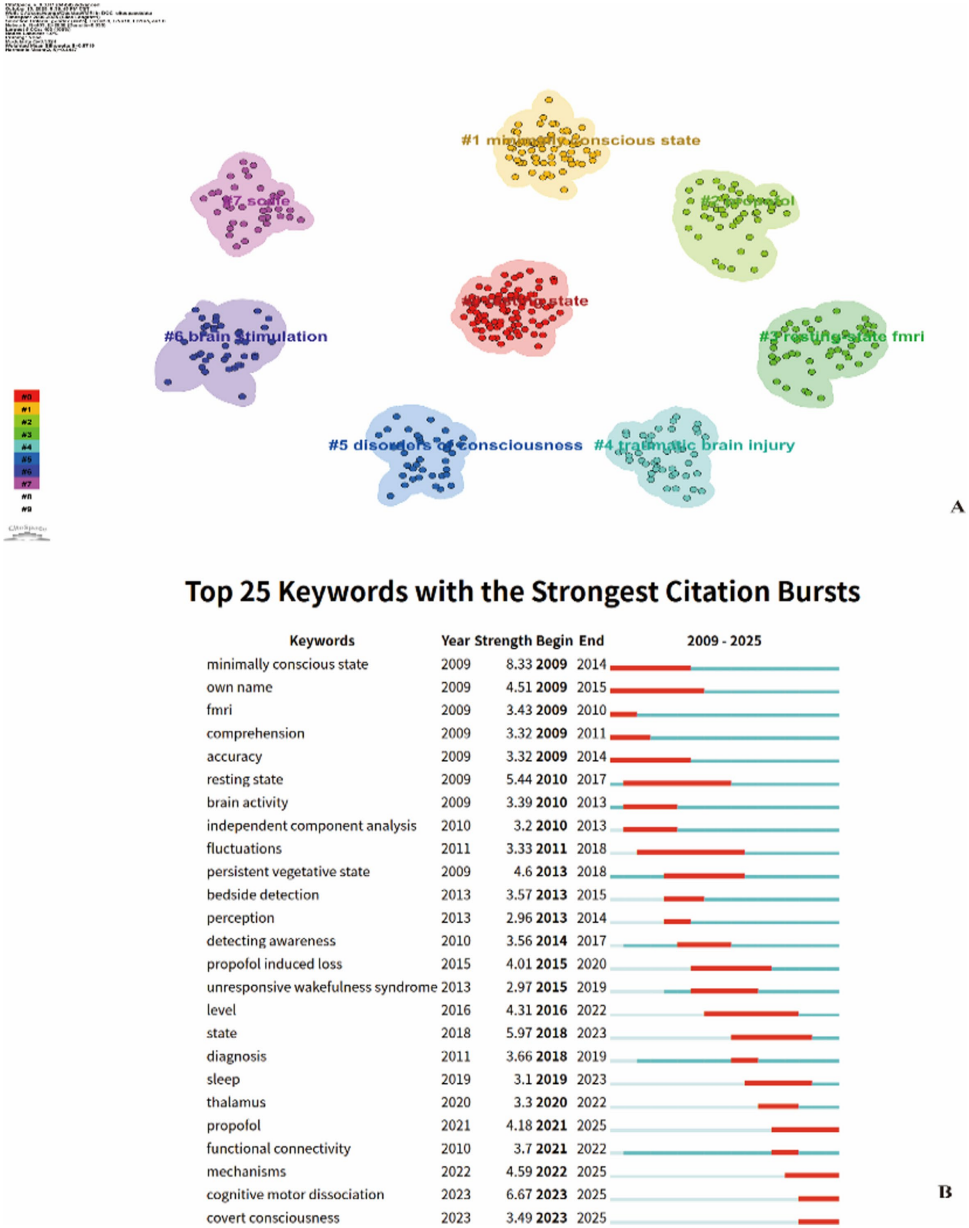
**(A)** Keywords Cluster view. The network map illustrates keyword co-occurrence relationships based on bibliometric data, with nodes representing individual keywords and edges indicating co-occurrence connections. Node size corresponds to keyword frequency, and colors denote distinct clusters identified through clustering algorithms: #0 minimally conscious state (red), #1 magnetic stimulation (yellow), #2 functional connectivity (green), #3 fMRI (light green), #4 traumatic brain injury (cyan), and #5 disorders of consciousness (blue). The clustering layout reveals six major research themes and their interconnections within the field of consciousness disorders from 2009 to 2025. **(B)** Top Keywords with the Strongest Citation Bursts Keyword burst analysis shows that keywords suddenly appear more frequently in the field within a certain period of time, and the red area indicates the length of time the keyword lasts from the beginning of the sudden appearance. (Software: CiteSpace 6.3. R1, Drexel University, Philadelphia, USA).

Co-occurrence network construction and cluster analysis of keywords identified 8 significant clusters (Modularity Q = 0.623, Silhouette mean = 0.78), categorized as follows: “# 0 resting state,” “#1 minimally conscious state,” “#2 propofol,” “#3 resting-state fMRI,” “#4 traumatic brain injury,” “#5 disorders of consciousness,” “#6 brain stimulation,” and “#7 scale” (Figure 6). Regarding evolutionary trajectories, both the temporal zone map and emergence analysis revealed that fMRI-related research initially emerged around 2010. Early keywords such as “resting state,” “fMRI,” and “brain activity” focused on method validation and consciousness state identification. Between 2013 and 2017, terms like “detecting awareness,” “persistent vegetative state,” and “bedside detection” emerged, indicating a gradual shift toward clinical translation and the feasibility of bedside detection. After 2020, “functional connectivity,” “thalamus,” and “covert consciousness” became the new wave of emergent keywords, marking the entry of fMRI research into the phase of identifying covert consciousness and analyzing its neural mechanisms, in particular attention now directed toward changes in functional connectivity within the thalamus, recognized as a critical node for consciousness maintenance. Mechanism research and intervention exploration.

**Figure 7:**
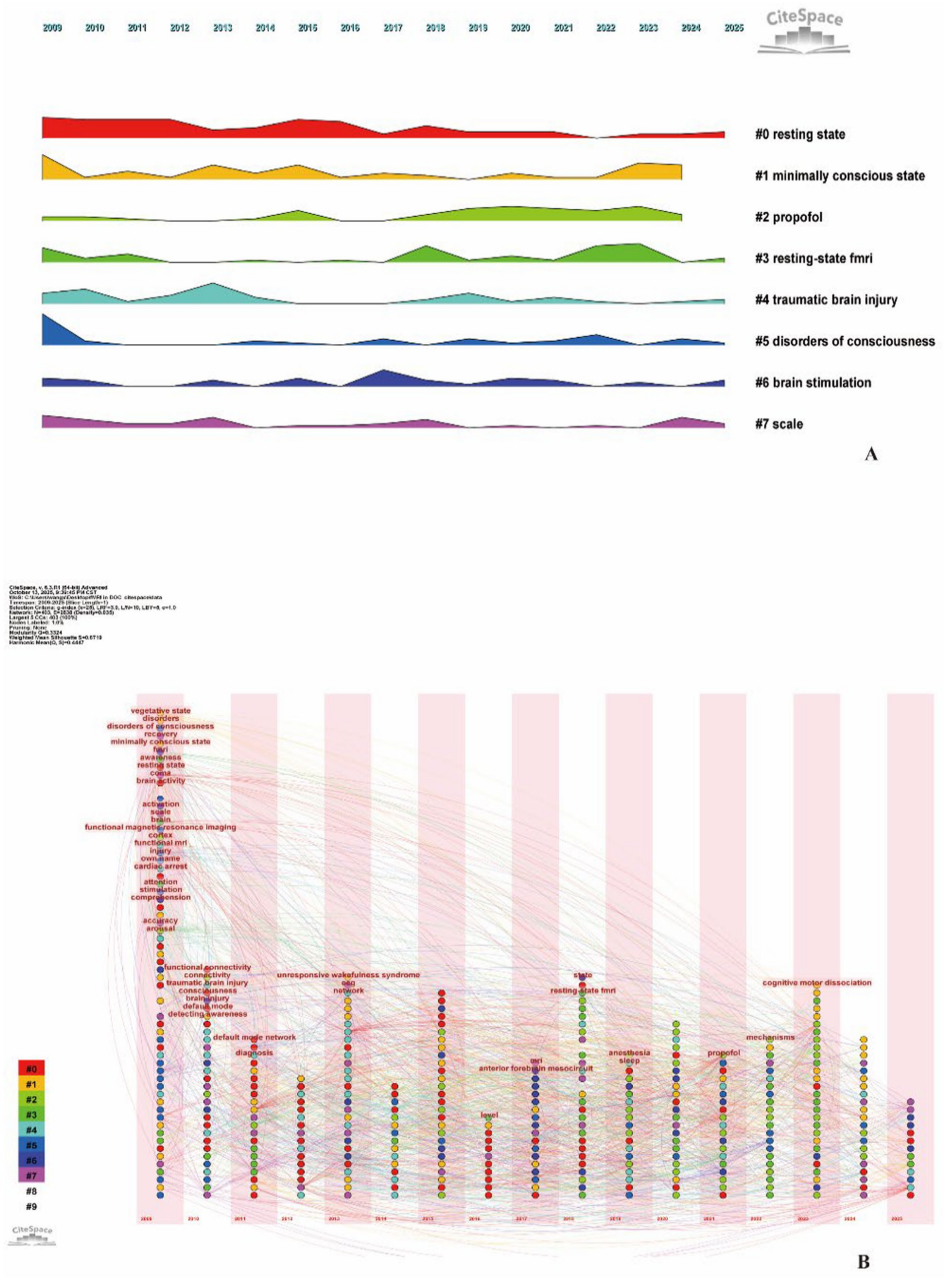
**(A)** Keywords landscape view, Temporal distribution of eight major keyword clusters from 2009 to 2025, showing the prominence and evolution of research themes over time. Clusters include #0 resting state, #1 minimally conscious state, #2 propofol, #3 resting-state fMRI, #4 traumatic brain injury, #5 disorders of consciousness, #6 brain stimulation, and #7 scale. **(B)** Keywords timezone view. Chronological progression of keyword emergence across clusters, with nodes representing keywords positioned by year of appearance. Colors denote cluster membership, and connecting lines indicate citation relationships, illustrating the developmental trajectory from foundational neuroimaging techniques to recent advances in covert consciousness detection.

Additionally, the landscape map (Figure 7) reveals that “resting-state fMRI” and “minimally conscious state” form high-frequency, high-centrality nodes at the core of the knowledge network. These nodes connect cutting-edge topics such as “brain stimulation,” “propofol,” and “cognitive-motor dissociation,” indicating that fMRI serves not only as a core diagnostic tool for pDoC but also as a pivotal technology linking mechanism research and intervention exploration.

## Discussion

fMRI serves as a crucial tool integrating early diagnosis, prognosis assessment, treatment decision-making, and ethical considerations, overcoming the limitations of traditional behavioral assessments by capturing neuronal cluster activity with high spatial resolution and providing clear neuroimaging evidence for determining the level of consciousness in patients with pDoC (20). This study conducted a bibliometric analysis of literature on fMRI diagnosis of pDoC, revealing a steady annual increase in published papers, with projections indicating a new peak by 2025. This demonstrates that research on fMRI for pDoC diagnosis is increasingly becoming a hot topic, driven by advancements in computer science and clinical imaging technologies.

Within the national collaboration network, the United States, China, and Belgium form the core nodes of global academic exchange. The United States holds a dominant position in the international cooperation system due to its publishing advantage and high bridging centrality (BC = 0.26). Although China ranks second in publication volume, its bridging centrality remains relatively limited (BC = 0.02), indicating a tendency for domestic collaboration among its academic teams. Future strategies should consider expanding transnational research, strengthening deep collaborative ties with cutting-edge research institutions, and enhancing international academic influence and global impact through high-quality scholarly outputs. At the institutional level, the University of Liège leads globally with 69 cumulative publications and a BC of 0.35, serving as a critical bridge between the EU, North America, and Asia. The university holds a prominent international position in the field of pDoC with its affiliated Coma Science Group and GIGA-Consciousness Research Center long focused on the pathological mechanisms, diagnostic strategies, and therapeutic interventions for DoC. Both organizations were co-founded by Professor Steven Laureys and his team members Olivia Gosseries and Aurore Thibaut. Author collaboration network analysis further indicates that Professor Laureys is a central node of academic influence in this field. Notably, Professor Laureys has been appointed as a full-time professor at the School of Basic Medicine, Hangzhou Normal University since 2025, leading the establishment of the “Zhejiang-Belgium Joint Laboratory on Consciousness Disorders.” Concurrently, he holds the position of Canada Excellence Research Chair at the Laval University Brain Research Center in Canada. His cross-regional academic appointments effectively promote knowledge exchange and resource integration between Chinese and Western research on consciousness disorders. Additionally, Professor Adrian M. Owen of Western University and Professor Joseph T. Giacino of Harvard University have made significant contributions in this field, forming a stable and close academic collaboration cluster with Professor Laureys.

In the co-citation network analysis of the literature, highly frequently co-cited papers reveal the methodological foundations and theoretical framework of the pDoC. Monti et al.’s landmark 2010 study “Willful modulation of brain activity in disorders of consciousness,” published in The New England Journal of Medicine, first demonstrated using fMRI that command-following brain activity modulation can detect covert consciousness in pDoC patients, establishing a critical objective biological marker for neurobehavioral assessment in this population. In the same year, Vanhaudenhuyse’s paper “Default network connectivity reflects the level of consciousness in a vegetative state,” published in Brain, systematically revealed a significant correlation between the strength of endogenous functional connectivity in the resting-state default network and the level of consciousness in VS patients. This provided core empirical evidence for the application of rs-fMRI in diagnostic stratification and pathological mechanism research for consciousness disorders. From the perspective of citation distribution at the journal level, Brain and The New England Journal of Medicine, leveraging their long-established academic authority, continue to play a central role in disseminating high-impact knowledge within this field.

The journal double-map overlay analysis (Figure 5) reveals that the citing journals on the left and the cited journals on the right span three major fields: neuroscience, computer systems science, and general internal medicine, establishing a comprehensive research framework encompassing “fundamental mechanisms-technical tools-clinical applications.” The “neuroscience → computer science → clinical medicine” pathway emerges as the primary research axis, characterized by high correlation (z = 4.791931) and high frequency (*f* = 78,312). Neuroscience lays the foundation for deciphering residual brain function mechanisms (e.g., default network activation). Computer technology propels fMRI from “qualitative description” (brain region activation) to “quantitative analysis” (functional connectivity strength, consciousness classification probability), overcoming the challenge of distinguishing UWS from MCS. Ultimately, this research “serves” clinical problem-solving and optimizes diagnostic and therapeutic approaches.

Based on keyword co-occurrence analysis, this study reveals research hotspots and future trends in fMRI applications for differential diagnosis of pDoC over the past two decades. The most frequently occurring keywords-“rs-fMRI,” “brain activity,” “detecting consciousness,” “bedside detection,” “functional connectivity,” “thalamus,” and “covert consciousness”-indicate that the literature primarily focuses on using bedside rs-fMRI to detect brain activity and functional connectivity for identifying covert consciousness in patients with pDoC. fMRI-related research emerged around 2010, focusing on using rs-fMRI to detect spontaneous brain activity in patients with pDoC. By analyzing activation in corresponding brain regions, researchers aimed to infer consciousness and cognitive states, thereby assessing levels of consciousness (21, 22). Unlike conventional fMRI, rs-fMRI does not require external stimulation, making it particularly useful for investigating functional brain networks in patients with pDoC (23). By detecting abnormal spontaneous brain activity in the cortical midline structures of patients with pDoC, rs-fMRI studies provided early empirical evidence for inferring consciousness levels and evaluating self-referential processing capacity using resting-state neuroimaging markers (24).

Between 2013 and 2017, keywords such as “persistent vegetative state,” “bedside detection,” and “detecting consciousness” emerged, indicating a shift toward clinical translation. The use of fMRI to assess residual consciousness in patients with pDoC holds significant clinical implications (25). It can fundamentally transform the diagnosis and understanding of patients’ cognitive status with profound ethical and medical implications, while also identifying covert brain activity to improve the accuracy of prognostic assessment, thereby providing families and healthcare providers with more precise information regarding patients’ rehabilitation potential and long-term outcomes (26, 27). A large-scale study on pDoC found that over two-thirds of unresponsive individuals later exhibited behavioral signs of consciousness recovery following the detection of covert consciousness through functional neuroimaging. However, fMRI techniques based on mental imagery tasks are challenging to implement, exhibit low diagnostic sensitivity, and demonstrate suboptimal accuracy in prognostic prediction, rendering them unsuitable as the primary imaging modality for consciousness disorder differentiation; they should serve only as supplementary approaches and be interpreted in conjunction with standardized behavioral assessments (13). Although fMRI has been recognized by several medical institutions, current guidelines do not recommend relying solely on fMRI results for clinical decision-making; instead, it should be integrated with multimodal data including CRS-R scale assessments, EEG, and evoked potentials (19).

Since 2020, emerging keywords such as “functional connectivity” and “thalamus” have transformed rs-fMRI from “voxel-wise activation maps” to “network-level biomarkers,” providing a multimodal validation framework for the differentiation of patients with pDoC. Gusnard and Raichle observed task-induced deactivations (negative BOLD responses) in the anterior cingulate cortex, posterior parietal cortex, and medial prefrontal cortex during resting state (28), while DMN functional connectivity has been shown to decrease with declining levels of consciousness (21, 22), and this deactivation is positively correlated with CRS-R scores (29, 30). A 35% reduction in posterior cingulate-medial prefrontal coupling strength quantitatively distinguished UWS from MCS (31). Posterior cingulate-precuneus connectivity independently predicted recovery of consciousness at 1 year with 81% accuracy, combined auditory paradigm improved accuracy to 91% (32, 33). Therefore, DMN functional connectivity not only demonstrates over 80% sensitivity in distinguishing MCS from UWS but also serves as an early predictor of consciousness recovery (33, 34). Diagnostic accuracy is further enhanced through cross-modal network integration. The visual and left frontoparietal networks, along with multi-network combined metrics, optimize classification performance (35), while external consciousness network connectivity may also correlate with levels of consciousness (15, 18, 33, 36). Global functional connectivity better distinguishes VS/UWS from MCS (37), dynamic correlation-anti-correlation network activity patterns can accurately classify MCS from VS/UWS (38), and 7 T high-resolution imaging enables assessment of subcortical hypothalamic-brainstem circuits (39–41). Dynamic functional connectivity further revealed that subclinical consciousness in CMD patients is characterized by reduced temporal stability in the Cingulo-Opercular Task Control Network, DMN, Fronto-Parietal Task Control Network, and Salience Network, providing new insights into consciousness mechanisms and the detection of covert consciousness in behaviorally non-responsive patients (42). Therefore, multi-network rs-fMRI not only enables precise identification of MCS but also provides a reliable imaging adjunct to bedside consciousness assessment (43). While rs-fMRI studies predominantly employ traditional machine learning to distinguish consciousness levels, graph theory and whole-brain computational modeling reveal pDoC network differences (44), yet precise differentiation among UWS, MCS, and CMD remains challenging. Yang et al.’s DeepDOC, based on 3D EfficientNet-B3, achieved an AUC of 0.93 for overall classification and an AUC of 1.0 for CMD identification using only rs-fMRI input, thereby delivering a task-free, interpretable, cross-center validated diagnostic framework through localization of cortical activation hotspots via gradient-weighted class activation mapping algorithm (45). A novel data processing workflow combining “low-dimensional latent space + interpretable neuro-glial models” simultaneously performs diagnostic classification and prognostic clustering at the individual patient level, directly generating personalized virtual brain models (46). Functional connectivity-based imaging-clinical fusion frameworks are progressively emerging as important complementary strategies for detecting consciousness levels, serving as adjuncts to traditional behavioral scales. Future efforts should deepen the integration of rs-fMRI with artificial intelligence, with the aim of achieving highly accurate, interpretable, and generalizable differential diagnosis of pDoC subgroups, ultimately facilitating the development of novel therapeutic interventions.

In 2023, “cognitive motor dissociation” and “covert consciousness” exhibited burst intensities of 6.67 and 3.49, respectively, emerging as the new frontiers in this field. Research into brain network mechanisms has driven rapid advances in fMRI, enabling clinical practice to leap from behavioral observation to circuit identification of implicit consciousness, leading to the recognition of CMD (12, 13, 47, 48). CMD is not a distinct novel state of consciousness, but rather represents misdiagnosed MCS + (minimally conscious state plus) patients, with short-term prognosis indistinguishable from MCS+ (49). CMD patients exhibit no behavioral responses during cognitive tasks (e.g., motor-imagery commands), yet show brain activation on fMRI (3, 11, 12, 50). In patients with preserved thalamocortical connectivity, 25% of behaviorally unresponsive patients are identified as CMD (51). Left thalamic lesions were present in 31% of non-CMD cases versus only 10% in CMD cases, supporting intact thalamic structure as a radiological criterion for CMD diagnosis (52). Traditional task-based methods may misclassify CMD patients as lacking conscious awareness due to impaired executive function, whereas rs-fMRI demonstrates increased temporal stability of dynamic functional connectivity, enabling independent CMD detection in the absence of commands (17, 42, 53). Task-based fMRI and EEG can increase the detection rate of CMD patients by an additional 10%, while multimodal assessments combining TMS-EEG substantially enhance diagnostic accuracy for patients with pDoC, thereby facilitating the development of more precise diagnostic protocols that integrate neuroimaging with electrophysiology (54).

“Resting-state fMRI” and “minimally conscious state” constitute high-frequency, high-centrality nodes positioned at the core of the knowledge network; the emergence of rs-fMRI has complemented bedside assessments and improved the accuracy of differentiating UWS from MCS. In 2002, the Aspen Neurobehavioral Working Group first introduced the concept of “MCS,” redefining the clinical spectrum of DOC and shifting the research focus from “behaviorally observable” to “mechanistically investigable” (55). Subsequent refinement of MCS into MCS + and MCS- poses greater challenges for behavior-based assessment of consciousness levels in pDoC patients (56), while accurate diagnosis impacts treatment, prognosis, and end-of-life decisions (3, 57–60). Early studies predominantly employed passive stimulation paradigms, in which subjects received various sensory inputs while fMRI monitored corresponding brain region activation. Twenty years ago, Schiff’s team presented auditory stimuli to two MCS patients, revealing cortical activity in the superior and middle temporal gyri comparable to that in healthy subjects (61). Subsequent auditory stimuli included name-calling, music, and emotionally charged movie clips (3, 62). Following brain injury, impaired processing of audiovisual stimuli, along with deficits in attention, memory, and executive function, leads to reduced efficacy of passive stimulation protocols that rely on sensory input, executive function, and motor output. Therefore, rs-fMRI capable of directly probing brain activity is required to identify biomarkers indicative of consciousness.

This high-frequency, high-centrality node further connects with frontier topics such as “brain stimulation” and “propofol,” quantitatively reflecting fMRI’s capacity to transform fundamental discoveries into clinical applications. The core mechanism of propofol-induced loss of consciousness lies in the selective inhibition of calbindin-rich thalamic matrix cells and their cross-modal cortical circuits, shifting the “core-matrix” functional architecture from a balanced state to a state of matrix disintegration, accompanied by significant disruption of functional connectivity within the DMN and between the DMN and Executive-Control Network (63); this quantifiable synergistic alteration in functional topology and network connectivity patterns provides a novel biomarker combining cellular and imaging characteristics for real-time diagnosis of patients with pDoC (64). The ultimate goal of stratifying patients with pDoC is not to determine “presence or absence of consciousness,” but to provide targetable neural network substrates and prognostic thresholds for personalized neuromodulation. fMRI plays a pivotal role in electrophysiological interventions: ① Maps thalamic subregion connectivity with 0.5 mm resolution to guide electrode implantation; ② Preserving baseline thalamic-frontal connectivity above 65% identifies CMD patients, enabling response stratification; ③ Real-time monitoring of DBS modulation on thalamic-cortical and cortical–cortical pathways via dynamic connectomics during and after surgery (65–67). Currently, large-scale multicenter randomized controlled trials on DBS intervention for patients with pDoC remain lacking, and optimal stimulation parameters (frequency, pulse width, duty cycle) alongside the plasticity time window remain undefined; future efforts should establish a “fMRI-electrophysiology-behavior” multimodal database and leverage deep learning to quantify DBS network modulation parameter-response relationships, thereby advancing fMRI as a critical decision-making reference for personalized neuromodulation in patients with pDoC.

### Clinical validation analysis: emerging therapeutic applications from PubMed

The following section presents qualitative narrative synthesis of PubMed-identified clinical trials (*n* = 18). No bibliometric algorithms were applied to these data. This analysis serves to contextualize WOSCC-identified research hotspots within recent clinical evidence, not to replicate or validate bibliometric findings through network analysis. The discussion is organized into four key areas. Research employing diffusion tensor imaging (DTI) has established a significant correlation between impaired white matter integrity and the level of consciousness, such as reduced fractional anisotropy values in patients with traumatic brain injury or hypoxic–ischemic encephalopathy (68, 69). By assessing connectivity within the DMN, rs-fMRI offers objective indicators for prognostic evaluation in post-cardiac arrest coma (70). Quantitative analysis of the apparent diffusion coefficient in diffusion-weighted imaging enables early prediction of neurological outcomes following out-of-hospital cardiac arrest (16, 71). Furthermore, multimodal image fusion techniques, including the combination of DTI and rs-fMRI, have improved diagnostic accuracy (72). Evidence suggests that the therapeutic effects of transcranial direct current stimulation and repetitive transcranial magnetic stimulation are closely linked to residual cerebral metabolism and gray matter integrity (73). Combined pharmacological interventions, such as amantadine with rTMS, can promote consciousness recovery in post-traumatic DoC patients by modulating thalamocortical circuits (1). Functional MRI-based neuronavigation also provides precise targeting for neuromodulation therapies (74). Pediatric patients with non-traumatic brain injury demonstrate distinct white matter injury patterns that differ significantly from those in adults (72). Diffuse white matter lesions observed in patients with COVID-19-associated prolonged coma reveal novel neuroinflammatory mechanisms following viral infection (75). In acute encephalopathy with diffuse bilateral hemispheric injury, specific associations exist between clinical manifestations and lesion topography (75). Common Data Elements for neuroimaging in disorders of consciousness have established a standardized framework for multicenter studies (76). Automated segmentation techniques for key brainstem nuclei now allow quantitative assessment of brainstem function (77). Finally, models of anesthesia-induced loss of consciousness, utilizing agents like propofol or midazolam, have elucidated neural mechanisms involving cortical–subcortical decoupling, providing a controlled experimental paradigm for consciousness research (78, 79).

This study represents the first systematic analysis using bibliometric methods to assess consciousness levels in patients with pDoC via fMRI, providing a more comprehensive insight into research hotspots and frontiers. However, this study has certain limitations. First, to ensure analysis quality, only English-language literature retrieved from the WOSCC was included. Consequently, the data sources are limited, and the exclusion of non-English literature, conference abstracts, and grey literature may result in data gaps and omit important studies. Second, this study primarily focuses on the value and significance of fMRI in diagnosing consciousness levels in pDoC patients, with limited coverage of its role in prognostic prediction and treatment-response evaluation. Multiple researchers participated in article screening to enhance accuracy and reduce bias. We explicitly clarify that WOSCC and PubMed data were analyzed separately using distinct methodologies-quantitative bibliometric analysis versus qualitative narrative review. This design does not permit cross-database validation of bibliometric patterns. Readers should interpret PubMed findings as clinical contextualization rather than empirical validation of WOSCC-derived hotspots. Future work aims to expand the search scope by incorporating international databases such as Scopus and Google Scholar. We plan to conduct clinical studies based on emerging trends while simultaneously developing a multidimensional knowledge graph linking “fMRI-intervention-outcome,” aiming to bridge the gap from bibliometric finding to evidence-based clinical decisions.

## Conclusion

This study systematically maps the evolution of fMRI for assessing pDoC from 2009 to 2025: since the emergence of resting-state techniques in 2010, both publication volume and academic quality in the field have increased in parallel. Research focus has shifted from “whether consciousness can be detected” to “how to accurately differentiate among levels?” Current clinical guidelines continue to rely exclusively on the CRS-R behavioral scale as the gold standard, lacking high-grade neuroimaging evidence. Therefore, this paper focuses on the value of rs-fMRI for assessment, the detection performance of CMD and covert consciousness, and the underlying thalamocortical network mechanisms. Integrating bibliometric findings indicates that constructing a targetable, predictable, and closed-loop imaging biomarker system has become the next frontier in pDoC diagnostics. fMRI will play an increasingly significant role in the diagnosis of pDoC: by integrating with multimodal data such as CRS-R and EEG, and employing advanced algorithms and personalized predictive models, its level of evidence is expected to continue rising, ultimately becoming the core evidence base for international clinical guidelines on consciousness disorders.

## Data Availability

The raw data supporting the conclusions of this article will be made available by the authors, without undue reservation.

## References

[1] Bender Pape TL Herrold AA Livengood SL Guernon A Weaver JA Higgins JP . A pilot trial examining the merits of combining amantadine and repetitive transcranial magnetic stimulation as an intervention for persons with disordered consciousness after TBI. J Head Trauma Rehabil. (2020) 35:371–87. doi: 10.1097/HTR.0000000000000634 33165151

[2] Kondziella D Stevens RD. Classifying disorders of consciousness: past, present, and future. Semin Neurol. (2022) 42:239–48. doi: 10.1055/a-1883-1021 35738291

[3] Edlow BL Claassen J Schiff ND Greer DM. Recovery from disorders of consciousness: mechanisms, prognosis and emerging therapies. Nat Rev Neurol. (2021) 17:135–56. doi: 10.1038/s41582-020-00428-x, 33318675 PMC7734616

[4] Kowalski RG Hammond FM Weintraub AH Nakase-Richardson R Zafonte RD Whyte J . Recovery of consciousness and functional outcome in moderate and severe traumatic brain injury. JAMA Neurol. (2021) 78:548–57. doi: 10.1001/jamaneurol.2021.0084 33646273 PMC7922241

[5] Wan X Zhang Y Li Y Song W. An update on noninvasive neuromodulation in the treatment of patients with prolonged disorders of consciousness. CNS Neurosci Ther. (2024) 30:e14757. doi: 10.1111/cns.14757, 38747078 PMC11094579

[6] Wang J Hu X Hu Z Sun Z Laureys S Di H. The misdiagnosis of prolonged disorders of consciousness by a clinical consensus compared with repeated coma-recovery scale-revised assessment. BMC Neurol. (2020) 20:343. doi: 10.1186/s12883-020-01924-9, 32919461 PMC7488705

[7] Monti MM Schnakers C. Flowchart for implementing advanced imaging and electrophysiology in patients with disorders of consciousness: to fMRI or not to fMRI? Neurology. (2022) 98:452–9. doi: 10.1212/WNL.0000000000200038 35058337

[8] Sala A Gosseries O Laureys S Annen J. Advances in neuroimaging in disorders of consciousness. Handb Clin Neurol. (2025) 207:97–127. doi: 10.1016/B978-0-443-13408-1.00008-7 39986730

[9] Kazazian K Monti MM Owen AM. Functional neuroimaging in disorders of consciousness: towards clinical implementation. Brain. (2025) 148:2283–98. doi: 10.1093/brain/awaf075, 39997570 PMC12233511

[10] Ogawa S Lee TM Kay AR Tank DW. Brain magnetic resonance imaging with contrast dependent on blood oxygenation. Proc Natl Acad Sci USA. (1990) 87:9868–72. doi: 10.1073/pnas.87.24.9868, 2124706 PMC55275

[11] Owen AM Coleman MR Boly M Davis MH Laureys S Pickard JD. Detecting Awareness in the Vegetative state. Science. (2006) 313:1402. doi: 10.1126/science.1130197 16959998

[12] Monti MM Vanhaudenhuyse A Coleman MR Boly M Pickard JD Tshibanda L . Willful modulation of brain activity in disorders of consciousness. N Engl J Med. (2010) 362:579–89. doi: 10.1056/NEJMoa0905370, 20130250

[13] Stender J Gosseries O Bruno M Charland-Verville V Vanhaudenhuyse A Demertzi A . Diagnostic precision of pet imaging and functional mri in disorders of consciousness: a clinical validation study. Lancet (London, England). (2014) 384:514–22. doi: 10.1016/S0140-6736(14)60042-8 24746174

[14] Zheng H Tian L Cai J. Meta-analysis of the diagnostic value of functional magnetic resonance imaging for distinguishing unresponsive wakefulness syndrome/vegetative state and minimally conscious state. Front Neurosci. (2024) 18:1395639. doi: 10.3389/fnins.2024.1395639 39315080 PMC11417101

[15] Medina JP Nigri A Stanziano M D'Incerti L Sattin D Ferraro S . Resting-state fmri in chronic patients with disorders of consciousness: the role of lower-order networks for clinical assessment. Brain Sci. (2022) 12:355. doi: 10.3390/brainsci12030355, 35326311 PMC8946756

[16] Khipal J Sankhyan N Singhi SC Singhi P Khandelwal N. Clinical utility of MRI brain in children with non-traumatic coma. Indian J Pediatr. (2017) 84:838–42. doi: 10.1007/s12098-017-2465-3 28936756

[17] Edlow BL Chatelle C Spencer CA Chu CJ Bodien YG O'Connor KL . Early detection of consciousness in patients with acute severe traumatic brain injury. Brain. (2017) 140:2399–414. doi: 10.1093/brain/awx176, 29050383 PMC6059097

[18] Martínez DE Rudas J Demertzi A Charland-Verville V Soddu A Laureys S . Reconfiguration of large-scale functional connectivity in patients with disorders of consciousness. Brain Behav. (2020) 10:e1476. doi: 10.1002/brb3.1476, 31773918 PMC6955826

[19] Giacino JT Katz DI Schiff ND Whyte J Ashman EJ Ashwal S . Practice guideline update recommendations summary: disorders of consciousness: report of the guideline development, dissemination, and implementation subcommittee of the american academy of neurology; the American congress of rehabilitation medicine; and the national institute on disability, independent living, and rehabilitation research. Arch Phys Med Rehabil. (2018) 99:1699–709. doi: 10.1016/j.apmr.2018.07.001 30098791

[20] Snider SB Edlow BL. Mri in disorders of consciousness. Curr Opin Neurol. (2020) 33:676–83. doi: 10.1097/WCO.0000000000000873 33044234 PMC8938962

[21] Boly M Tshibanda L Vanhaudenhuyse A Noirhomme Q Schnakers C Ledoux D . Functional connectivity in the default network during resting state is preserved in a vegetative but not in a brain dead patient. Hum Brain Mapp. (2009) 30:2393–400. doi: 10.1002/hbm.20672, 19350563 PMC6870763

[22] Vanhaudenhuyse A Noirhomme Q Tshibanda LJ Bruno M Boveroux P Schnakers C . Default network connectivity reflects the level of consciousness in non-communicative brain-damaged patients. Brain. (2010) 133:161–71. doi: 10.1093/brain/awp313 20034928 PMC2801329

[23] Schwarzbauer C Schafer B. fMRI in disorders of consciousness: future diagnostic opportunities, methodological and legal challenges. Cortex. (2011) 47:1243–5. doi: 10.1016/j.cortex.2011.04.014 21612771

[24] Huang Z Dai R Wu X Yang Z Liu D Hu J . The self and its resting state in consciousness: an investigation of the vegetative state. Hum Brain Mapp. (2014) 35:1997–2008. doi: 10.1002/hbm.22308 23818102 PMC6868996

[25] Fernández-Espejo D Owen AM. Detecting awareness after severe brain injury. Nat Rev Neurosci. (2013) 14:801–9. doi: 10.1038/nrn3608 24088810

[26] Coleman MR Davis MH Rodd JM Robson T Ali A Owen AM . Towards the routine use of brain imaging to aid the clinical diagnosis of disorders of consciousness. Brain. (2009) 132:2541–52. doi: 10.1093/brain/awp183 19710182

[27] Gui P Jiang Y Zang D Qi Z Tan J Tanigawa H . Assessing the depth of language processing in patients with disorders of consciousness. Nat Neurosci. (2020) 23:761–70. doi: 10.1038/s41593-020-0639-1, 32451482

[28] Gusnard DA Raichle ME Raichle ME. Searching for a baseline: functional imaging and the resting human brain. Nat Rev Neurosci. (2001) 2:685–94. doi: 10.1038/35094500 11584306

[29] Estraneo A Moretta P Loreto V Santoro L Trojano L. Clinical and neuropsychological long-term outcomes after late recovery of responsiveness: a case series. Arch Phys Med Rehabil. (2014) 95:711–6. doi: 10.1016/j.apmr.2013.11.004 24275063

[30] Crone JS Ladurner G Höller Y Golaszewski S Trinka E Kronbichler M. Deactivation of the default mode network as a marker of impaired consciousness: an fMRI study. PLoS One. (2011) 6:e26373. doi: 10.1371/journal.pone.0026373 22039473 PMC3198462

[31] Soddu A Vanhaudenhuyse A Bahri MA Bruno M Boly M Demertzi A . Identifying the default-mode component in spatial ic analyses of patients with disorders of consciousness. Hum Brain Mapp. (2012) 33:778–96. doi: 10.1002/hbm.21249 21484953 PMC6870518

[32] Wu X Zou Q Hu J Tang W Mao Y Gao L . Intrinsic functional connectivity patterns predict consciousness level and recovery outcome in acquired brain injury. J Neurosci. (2015) 35:12932–46. doi: 10.1523/JNEUROSCI.0415-15.2015 26377477 PMC4571611

[33] Demertzi A Antonopoulos G Heine L Voss HU Crone JS de Los AC . Intrinsic functional connectivity differentiates minimally conscious from unresponsive patients. Brain. (2015) 138:2619–31. doi: 10.1093/brain/awv169 26117367

[34] Demertzi A Gómez F Crone JS Vanhaudenhuyse A Tshibanda L Noirhomme Q . Multiple fMRI system-level baseline connectivity is disrupted in patients with consciousness alterations. Cortex. (2014) 52:35–46. doi: 10.1016/j.cortex.2013.11.005 24480455

[35] Seitzman BA Snyder AZ Leuthardt EC Shimony JS. The state of resting state networks. Top Magnet Resonance Imag. (2019) 28:189–96. doi: 10.1097/RMR.0000000000000214, 31385898 PMC6686880

[36] Liang X Zou Q He Y Yang Y. Topologically reorganized connectivity architecture of default-mode, executive-control, and salience networks across working memory task loads. Cereb Cortex. (2016) 26:1501–11. doi: 10.1093/cercor/bhu316, 25596593 PMC4785946

[37] Kotchoubey B Merz S Lang S Markl A Müller F Yu T . Global functional connectivity reveals highly significant differences between the vegetative and the minimally conscious state. J Neurol. (2013) 260:975–83. doi: 10.1007/s00415-012-6734-9, 23128970

[38] Demertzi A Tagliazucchi E Dehaene S Deco G Barttfeld P Raimondo F . Human consciousness is supported by dynamic complex patterns of brain signal coordination. Sci Adv. (2019) 5:eaat7603. doi: 10.1126/sciadv.aat7603, 30775433 PMC6365115

[39] Beissner F Schumann A Brunn F Eisenträger D Bär K. Advances in functional magnetic resonance imaging of the human brainstem. NeuroImage. (2014) 86:91–8. doi: 10.1016/j.neuroimage.2013.07.081 23933038

[40] Bianciardi M Toschi N Eichner C Polimeni JR Setsompop K Brown EN . In vivo functional connectome of human brainstem nuclei of the ascending arousal, autonomic, and motor systems by high spatial resolution 7-tesla fmri. MAGMA. (2016) 29:451–62. doi: 10.1007/s10334-016-0546-3, 27126248 PMC4892960

[41] Bär K de la Cruz F Schumann A Koehler S Sauer H Critchley H . Functional connectivity and network analysis of midbrain and brainstem nuclei. NeuroImage. (2016) 134:53–63. doi: 10.1016/j.neuroimage.2016.03.071, 27046112

[42] Wu H Xie Q Pan J Liang Q Lan Y Guo Y . Identifying patients with cognitive motor dissociation using resting-state temporal stability. NeuroImage. (2023) 272:120050. doi: 10.1016/j.neuroimage.2023.120050, 36963740

[43] Medina Carrion JP Stanziano M D'Incerti L Sattin D Ferraro S Rossi Sebastiano D . Detecting rs-fMRI networks in disorders of consciousness: improving clinical interpretability. Ann Clin Transl Neurol. (2025) 12:1771–84. doi: 10.1002/acn3.70094, 40577112 PMC12455880

[44] Campbell JM Huang Z Zhang J Wu X Qin P Northoff G . Pharmacologically informed machine learning approach for identifying pathological states of unconsciousness via resting-state fMRI. NeuroImage. (2020) 206:116316. doi: 10.1016/j.neuroimage.2019.116316, 31672663 PMC6981054

[45] Yang H Wu H Kong L Luo W Xie Q Pan J . Precise detection of awareness in disorders of consciousness using deep learning framework. NeuroImage. (2024) 290:120580. doi: 10.1016/j.neuroimage.2024.120580, 38508294

[46] Zonca L Escrichs A Patow G Manasova D Sanz-Perl Y Annen J . Personalized models of disorders of consciousness reveal complementary roles of connectivity and local parameters in diagnosis and prognosis. PLoS One. (2025) 20:e328219. doi: 10.1371/journal.pone.0328219, 40892891 PMC12404413

[47] Cruse D Chennu S Chatelle C Bekinschtein TA Fernández-Espejo D Pickard JD . Bedside detection of awareness in the vegetative state: a cohort study. Lancet (London, England). (2011) 378:2088–94. doi: 10.1016/S0140-6736(11)61224-5, 22078855

[48] Curley WH Forgacs PB Voss HU Conte MM Schiff ND. Characterization of eeg signals revealing covert cognition in the injured brain. Brain. (2018) 141:1404–21. doi: 10.1093/brain/awy070, 29562312 PMC5917770

[49] Monti MM. Is cognitive motor dissociation just a minimally conscious state "plus" by another name? [Epub ahead of print] (2025). doi: 10.1101/2025.06.11.25329346.

[50] Schiff ND. Cognitive motor dissociation following severe brain injuries. JAMA Neurol. (2015) 72:1413–5. doi: 10.1001/jamaneurol.2015.2899 26502348

[51] Bodien YG Allanson J Cardone P Bonhomme A Carmona J Chatelle C . Cognitive motor dissociation in disorders of consciousness. N Engl J Med. (2024) 391:598–608. doi: 10.1056/NEJMoa2400645, 39141852 PMC7617195

[52] Franzova E Shen Q Doyle K Chen JM Egbebike J Vrosgou A . Injury patterns associated with cognitive motor dissociation. Brain. (2023) 146:4645–58. doi: 10.1093/brain/awad197, 37574216 PMC10629765

[53] Sanz LRD Thibaut A Edlow BL Laureys S Gosseries O. Update on neuroimaging in disorders of consciousness. Curr Opin Neurol. (2021) 34:488–96. doi: 10.1097/WCO.0000000000000951, 34054109 PMC8938964

[54] Lo CCH Woo PYM Cheung VCK. Task-based eeg and fmri paradigms in a multimodal clinical diagnostic framework for disorders of consciousness. Rev Neurosci. (2024) 35:775–87. doi: 10.1515/revneuro-2023-0159 38804042

[55] Giacino JT Ashwal S Childs N Cranford R Jennett B Katz DI . The minimally conscious state: definition and diagnostic criteria. Neurology. (2002) 58:349–53. doi: 10.1212/WNL.58.3.349, 11839831

[56] Bruno M Vanhaudenhuyse A Thibaut A Moonen G Laureys S. From unresponsive wakefulness to minimally conscious plus and functional locked-in syndromes: recent advances in our understanding of disorders of consciousness. J Neurol. (2011) 258:1373–84. doi: 10.1007/s00415-011-6114-x 21674197

[57] Dolce G Quintieri M Serra S Lagani V Pignolo L. Clinical signs and early prognosis in vegetative state: a decisional tree, data-mining study. Brain Inj. (2008) 22:617–23. doi: 10.1080/02699050802132503, 18568716

[58] Faugeras F Rohaut B Valente M Sitt J Demeret S Bolgert F . Survival and consciousness recovery are better in the minimally conscious state than in the vegetative state. Brain Inj. (2018) 32:72–7. doi: 10.1080/02699052.2017.1364421 29156989

[59] Hirschberg R Giacino JT. The vegetative and minimally conscious states: diagnosis, prognosis and treatment. Neurol Clin. (2011) 29:773–86. doi: 10.1016/j.ncl.2011.07.009 22032660

[60] Luauté J Maucort-Boulch D Tell L Quelard F Sarraf T Iwaz J . Long-term outcomes of chronic minimally conscious and vegetative states. Neurology. (2010) 75:246–52. doi: 10.1212/WNL.0b013e3181e8e8df, 20554940

[61] Schiff ND Rodriguez-Moreno D Kamal A Kim KHS Giacino JT Plum F . Fmri reveals large-scale network activation in minimally conscious patients. Neurology. (2005) 64:514–23. doi: 10.1212/01.WNL.0000150883.10285.44, 15699384

[62] Naci L Cusack R Anello M Owen AM. A common neural code for similar conscious experiences in different individuals. Proc Natl Acad Sci USA. (2014) 111:14277–82. doi: 10.1073/pnas.1407007111, 25225384 PMC4191782

[63] Boveroux P Vanhaudenhuyse A Bruno M Noirhomme Q Lauwick S Luxen A . Breakdown of within- and between-network resting state functional magnetic resonance imaging connectivity during propofol-induced loss of consciousness. Anesthesiology. (2010) 113:1038–53. doi: 10.1097/ALN.0b013e3181f697f5, 20885292

[64] Huang Z Mashour GA Hudetz AG. Propofol disrupts the functional core-matrix architecture of the thalamus in humans. Nat Commun. (2024) 15:7496. doi: 10.1038/s41467-024-51837-1, 39251579 PMC11384736

[65] Zhang D Snyder AZ Fox MD Sansbury MW Shimony JS Raichle ME. Intrinsic functional relations between human cerebral cortex and thalamus. J Neurophysiol. (2008) 100:1740–8. doi: 10.1152/jn.90463.2008, 18701759 PMC2576214

[66] Kumar VJ van Oort E Scheffler K Beckmann CF Grodd W. Functional anatomy of the human thalamus at rest. NeuroImage. (2017) 147:678–91. doi: 10.1016/j.neuroimage.2016.12.071 28041978

[67] Tian Y Margulies DS Breakspear M Zalesky A. Topographic organization of the human subcortex unveiled with functional connectivity gradients. Nat Neurosci. (2020) 23:1421–32. doi: 10.1038/s41593-020-00711-6 32989295

[68] van der Eerden AW Khalilzadeh O Perlbarg V Dinkel J Sanchez P Vos PE . White matter changes in comatose survivors of anoxic ischemic encephalopathy and traumatic brain injury: comparative diffusion-tensor imaging study. Radiology. (2014) 270:506–16. doi: 10.1148/radiol.13122720, 24471392

[69] Wilde EA Li X Hunter JV Narayana PA Hasan K Biekman B . Loss of consciousness is related to white matter injury in mild traumatic brain injury. J Neurotrauma. (2016) 33:2000–10. doi: 10.1089/neu.2015.4212, 26801471

[70] Shao R Wang T Hang C An L Wang X Zhang L . Alteration in early resting-state functional MRI activity in comatose survivors of cardiac arrest: a prospective cohort study. Crit Care. (2024) 28:260. doi: 10.1186/s13054-024-05045-4, 39095884 PMC11295486

[71] Yoon JA Kang C Park JS You Y Min JH In YN . Quantitative analysis of early apparent diffusion coefficient values from MRIs for predicting neurological prognosis in survivors of out-of-hospital cardiac arrest: an observational study. Crit Care. (2023) 27:407. doi: 10.1186/s13054-023-04696-z, 37880777 PMC10599006

[72] Molteni E Rocca MA Strazzer S Pagani E Colombo K Arrigoni F . A diffusion tensor magnetic resonance imaging study of paediatric patients with severe non-traumatic brain injury. Dev Med Child Neurol. (2017) 59:199–206. doi: 10.1111/dmcn.13332, 27910995

[73] Thibaut A Di Perri C Chatelle C Bruno M Bahri MA Wannez S . Clinical response to tdcs depends on residual brain metabolism and grey matter integrity in patients with minimally conscious state. Brain Stimul. (2015) 8:1116–23. doi: 10.1016/j.brs.2015.07.024 26471400

[74] Magrassi L Maggioni G Pistarini C Di Perri C Bastianello S Zippo AG . Results of a prospective study (cats) on the effects of thalamic stimulation in minimally conscious and vegetative state patients. J Neurosurg. (2016) 125:972–81. doi: 10.3171/2015.7.JNS15700 26745476

[75] Abdo WF Broerse CI Grady BP Wertenbroek AAAC Vijlbrief O Buise MP . Prolonged unconsciousness following severe covid-19. Neurology. (2021) 96:e1437–42. doi: 10.1212/WNL.0000000000011355, 33443134 PMC8055315

[76] Edlow BL Boerwinkle VL Annen J Boly M Gosseries O Laureys S . Common data elements for disorders of consciousness: recommendations from the working group on neuroimaging. Neurocrit Care. (2023) 39:611–7. doi: 10.1007/s12028-023-01794-2, 37552410

[77] Olchanyi MD Augustinack J Haynes RL Lewis LD Cicero N Li J . Automated mri segmentation of brainstem nuclei critical to consciousness. Hum Brain Mapp. (2025) 46:e70357. doi: 10.1002/hbm.70357, 41074651 PMC12514455

[78] Ferrarelli F Massimini M Sarasso S Casali A Riedner BA Angelini G . Breakdown in cortical effective connectivity during midazolam-induced loss of consciousness. Proc Natl Acad Sci USA. (2010) 107:2681–6. doi: 10.1073/pnas.0913008107, 20133802 PMC2823915

[79] Fernández-Candil JL Nuttall R Gallart L Schneider G Blanco-Hinojo L Martínez-Vilavella G . Electroencephalographic changes related to cortico-subcortical decoupling during propofol-induced loss of consciousness: a secondary analysis of a prospective observational study. J Clin Anesth. (2025) 106:111926. doi: 10.1016/j.jclinane.2025.111926, 40682867

