# Supplementary material for: Mapping fMRI research in disorders of consciousness: a bibliometric study
Source: Front Neurol. 2026 May 4;17:1807532. doi: 10.3389/fneur.2026.1807532 (PMC13180544; doi:10.3389/fneur.2026.1807532)
Supplement: Supplementary file 1 [file Table_1.doc]

(((("Consciousness Disorders"[Mesh] OR "Persistent Vegetative State"[Mesh] OR "Minimally Conscious State"[Mesh] OR "Unconsciousness"[Mesh]) OR (persistent vegetative state [Title/Abstract] OR vegetative state* [Title/Abstract] OR consciousness disorder* [Title/Abstract] OR unawareness state* [Title/Abstract] OR disorders of consciousness [Title/Abstract] OR prolonged loss of consciousness [Title/Abstract] OR prolonged unconscious state* [Title/Abstract] OR minimally conscious state* [Title/Abstract] OR minimal conscious state* [Title/Abstract] OR unresponsive wakefulness syndrome [Title/Abstract] OR UWS [Title/Abstract] OR cognitive-motor dissociation [Title/Abstract] OR (CMD [Title/Abstract] AND (consciousness [Title/Abstract] OR vegetative [Title/Abstract]))))) AND ((("Magnetic Resonance Imaging"[Mesh] OR "Brain Mapping"[Mesh]) OR (fMRI [Title/Abstract] OR functional magnetic resonance imaging [Title/Abstract] OR functional MRI [Title/Abstract] OR resting-state functional magnetic resonance imaging [Title/Abstract] OR rs-fMRI [Title/Abstract] OR resting-state fMRI [Title/Abstract] OR task-functional magnetic resonance imaging [Title/Abstract] OR task-fMRI [Title/Abstract])))) AND ("Journal Article"[Publication Type] AND Humans[Mesh] AND 2009:2025[Date - Publication]

Article type：Clinical Study, Clinical Trial, Controlled Clinical Trial, Multicenter Study, Observational Study, Randomized Controlled Trial
