# Supplementary material for: Mapping fMRI research in disorders of consciousness: a bibliometric study
Source: Front Neurol. 2026 May 4;17:1807532. doi: 10.3389/fneur.2026.1807532 (PMC13180544; doi:10.3389/fneur.2026.1807532)
Supplement: Supplementary file 5 [file Table_5.doc]

| Search strategy from Web of Science Core Collection | |
| --- | --- |
| Set | Workflows |
| #3 | #1AND#2 |
| #1 | (TS=(persistent vegetative state OR vegetative state* OR consciousness disorder* OR unawareness state* OR disorders of consciousness OR prolonged loss of consciousness OR prolonged unconscious state* OR minimally conscious state* OR minimal conscious state* OR unresponsive wakefulness syndrome OR UWS OR cognitive-motor dissociation OR CMD)) |
| #2 | (TS=(fMRI OR functional magnetic resonance imaging OR functional MRI OR resting-state functional magnetic resonance imaging OR rs-fMRI OR resting-state fMRI OR task-functional magnetic resonance imaging OR task-fMRI )) |

TS: topic

Date of search:From 2009 to September 11, 2025

Article type: Article or Review
